# Construction of a reference genetic linkage map for carnation (*Dianthus caryophyllus* L*.*)

**DOI:** 10.1186/1471-2164-14-734

**Published:** 2013-10-26

**Authors:** Masafumi Yagi, Toshiya Yamamoto, Sachiko Isobe, Hideki Hirakawa, Satoshi Tabata, Koji Tanase, Hiroyasu Yamaguchi, Takashi Onozaki

**Affiliations:** 1 NARO Institute of Floricultural Science (NIFS), 2-1 Fujimoto, Tsukuba, Ibaraki 305-8519, Japan; 2 NARO Institute of Fruit Tree Science (NIFTS), 2-1 Fujimoto, Tsukuba, Ibaraki 305-8605, Japan; 3 Kazusa DNA Research Institute, 2-6-7 Kazusa-kamatari, Kisarazu, Chiba 292-0818, Japan

**Keywords:** Carnation, *Dianthus caryophyllus* L, EST, Linkage map, Next-generation sequencing technology (NGS), RAPD, STS, SSR

## Abstract

**Background:**

Genetic linkage maps are important tools for many genetic applications including mapping of quantitative trait loci (QTLs), identifying DNA markers for fingerprinting, and map-based gene cloning. Carnation (*Dianthus caryophyllus* L.) is an important ornamental flower worldwide. We previously reported a random amplified polymorphic DNA (RAPD)-based genetic linkage map derived from *Dianthus capitatus* ssp. *andrezejowskianus* and a simple sequence repeat (SSR)-based genetic linkage map constructed using data from intraspecific F_2_ populations; however, the number of markers was insufficient, and so the number of linkage groups (LGs) did not coincide with the number of chromosomes (x = 15). Therefore, we aimed to produce a high-density genetic map to improve its usefulness for breeding purposes and genetic research.

**Results:**

We improved the SSR-based genetic linkage map using SSR markers derived from a genomic library, expression sequence tags, and RNA-seq data. Linkage analysis revealed that 412 SSR loci (including 234 newly developed SSR loci) could be mapped to 17 linkage groups (LGs) covering 969.6 cM. Comparison of five minor LGs covering less than 50 cM with LGs in our previous RAPD-based genetic map suggested that four LGs could be integrated into two LGs by anchoring common SSR loci. Consequently, the number of LGs corresponded to the number of chromosomes (x = 15). We added 192 new SSRs, eight RAPD, and two sequence-tagged site loci to refine the RAPD-based genetic linkage map, which comprised 15 LGs consisting of 348 loci covering 978.3 cM. The two maps had 125 SSR loci in common, and most of the positions of markers were conserved between them. We identified 635 loci in carnation using the two linkage maps. We also mapped QTLs for two traits (bacterial wilt resistance and anthocyanin pigmentation in the flower) and a phenotypic locus for flower-type by analyzing previously reported genotype and phenotype data.

**Conclusions:**

The improved genetic linkage maps and SSR markers developed in this study will serve as reference genetic linkage maps for members of the genus *Dianthus*, including carnation, and will be useful for mapping QTLs associated with various traits, and for improving carnation breeding programs.

## Background

Genetic linkage maps are valuable resources that provide a framework for many genomic analyses. They are an important tool for many genetic applications, including mapping of quantitative trait loci (QTLs), comparative mapping, identifying DNA markers for fingerprinting, analyses of population genetics and phylogenetics, genome sequence assembly, relating physical and recombination distances along the genome, and map-based cloning of genes. The wide applications of linkage maps and their importance in genetic research has led to numerous linkage mapping projects being undertaken in plants [1-3].

Carnation (*Dianthus caryophyllus* L.), in the Caryophyllaceae, is one of the major floricultural crops in Japan and around the world. Hundreds of cultivars are grown around the world and many new varieties are bred and registered every year [4]. More than 300 *Dianthus* species have been recorded [5]. Many *Dianthus* species are distributed throughout Europe and Asia, and the distribution of the genus extends from arctic North America to mountainous sites in Africa [6]. High-quality commercial carnation cultivars are usually obtained by inter- or intraspecific hybridization [7-9].

Most of the carnation cultivars are diploid with a chromosome number of 2n = 2x = 30 [7]. The reported nuclear DNA content of carnation is 1.23–1.48 pg/2C [10-12]. Therefore, carnation has a comparatively small nuclear genome (minimum estimate, 611 Mb) approximately four times the size of the *Arabidopsis thaliana* genome (0.30 pg/2C) [13]. According to the Plant C-values database (http://data.kew.org/cvalues/), the genome of carnation is very small compared with those of other ornamental flowers such as *Rosa hybrida* (1.1 Gb), *Antirrhinum majus* (1.5 Gb), *Petunia hybrida* (1.6 Gb), *Chrysanthemum morifolium* (9.4 Gb), and *Tulipa gesneriana* (26 Gb).

To improve the selection efficiency in breeding plants with resistance to carnation bacterial wilt (CBW) using *D. capitatus* ssp. *andrezejowskianus*, we constructed the first genetic linkage map for carnation, which comprised 137 random amplified polymorphic DNA (RAPD) and nine simple sequence repeat (SSR) loci within 16 linkage groups (LGs) [14] (the “A” map in Yagi et al. [15]). A QTL analysis identified one major QTL (*Cbw1*) and two minor QTLs (*Cbw2* and *Cbw3*) for CBW resistance derived from *D. capitatus* ssp. *andrezejowskianus*. We also identified a new QTL for CBW resistance (*Cbw4*), derived from carnation line 85–11, using an SSR-based genetic linkage map (the “B” map in Yagi et al. [15]). The “B’ map comprised 178 SSR loci, covering 843.6 cM with an average distance of 6.5 cM between loci, and 16 LGs. This was the first report of a genetic linkage map based mainly on SSR markers for the genus *Dianthus*. The SSR markers and the genetic linkage maps developed for carnation in our previous studies would be useful for comparisons between mapping populations, for QTL mapping of important agronomic characteristics, and for phylogenetic analyses. However, the number of markers was insufficient for these purposes. Also, the small number of markers had resulted in 16 LGs being identified on the maps, instead of 15 (the actual number of chromosomes in carnation). We conducted RNA-seq analysis and obtained 300,740 unigenes consisting of 37,844 contigs and 262,896 singletons using next-generation sequencing (NGS) technology [16]. We identified 17,362 potential SSRs in 14,291 of the unigenes. Finally, 4,177 SSR primer pairs were designed [16].

In this study, we aimed to construct a high-density SSR-based genetic linkage map to serve as a reference for members of *Dianthus*. We evaluated polymorphisms in the mapping population (the “B” map) using SSR primer pairs designed from data from the RNA-seq analysis, the genomic SSR library, and Sanger-sequencing expressed sequence tag (EST) analysis [15]. We refined the “A” map by adding the new RAPD and SSR loci. Comparative analysis of the refined “A” and “B” maps revealed corresponding LGs between the two linkage maps. Also, we analyzed previously reported genotype and phenotype data to map two QTLs (for CBW resistance and flower anthocyanin pigmentation) and a flower-type locus onto the refined maps.

## Results

### Genotyping of the 85P population

The name of the mapping population was changed from “B” to “85P” in this study. Screening of 1,013 SSR primer pairs (beginning with the letters “GS”) derived from the SSR-enriched genomic libraries [15] revealed 62 new “GS” SSR markers that were polymorphic in 85P progenies (Table 1). Therefore, a total of 121 “GS” SSR markers, including 59 reported previously [15], were polymorphic in the 85P population. We reanalyzed 1,530 “CES” primer pairs derived from EST analysis [15] using fluorescent fragment analysis instead of electrophoresis on polyacrylamide gels. This analysis revealed 65 new polymorphic “CES” markers (Table 1). Therefore, a total of 174 “CES” SSR markers including 109 markers reported previously [15] were polymorphic in the 85P population. Tanase et al. [16] designed 4,177 SSR primer pairs from 17,362 potential SSR repeats by RNA-seq analysis. The names of these SSR markers begin with the letters “NES”. Of 897 “NES” SSR primers that included di- and tri- nucleotide SSR motifs, 387 produced amplicons that were not polymorphic between parents, 403 produced no amplicon, weak amplicons, or multi-locus amplified products, and 107 produced amplicons that were polymorphic (Table 1). Additional file 1 shows the newly developed SSR markers for the 85P map.

**Table 1:** Number and type of loci, genetic distance and marker density for refined 85P map

**New LG**	**Previous LG (Yagi et al. **[15]**)**	**Total No. of SSR loci**	**Mapped SSR loci (in Yagi et al. **[15]**)**	**New SSR loci**				**Length of LGs(cM)**	**Marker density (loci/cM)**	**Common SSR loci between 85P and NP map**
				**Locus names**			**Subtotal**			
				**GS-**	**CES-**	**NES-**				
			**SSR resource**							
			**Genomic, EST**	**Genomic**	**EST**	**EST**				
85P_1	B1	48	17	10	9	12	31	111.9	0.43	12
85P_2	B2	16	9	1	3	3	7	97.1	0.16	7
85P_3	B3	38	17	4	7	10	21	70.2	0.54	10
85P_4	B4	39	21	9	7	2	18	73.9	0.53	14
85P_5	B5	24	10	4	2	8	14	64.6	0.37	4
85P_6	B6	29	18	1	5	5	11	72.1	0.40	5
85P_7	B7	27	12	6	1	8	15	66.2	0.41	7
85P_8	B8	36	18	6	4	8	18	66.3	0.54	8
85P_9	B9	25	9	4	2	10	16	65.1	0.38	3
85P_10	B10	32	10	3	7	12	22	56.1	0.57	6
85P_11	B11	19	9	2	3	5	10	81.6	0.23	4
85P_12	B12	28	9	4	7	8	19	52.1	0.54	8
85P_13	B13	18	7	3	2	6	11	33.0	0.55	13
85P_14-1	B14	7	3	2	1	1	4	23.2	0.30	6
85P_14-2	B16	7	2	0	0	5	5	18.4	0.38	5
85P_15-1	B15	13	5	1	4	3	8	10.1	1.29	11
85P_15-2	no linkage group	6	2	2	1	1	4	7.7	0.78	2
Total		412	178	62	65	107	234	969.6	0.42	125

We identified 412 SSR markers that showed single-locus polymorphisms in our mapping population, including 10 SSR markers mapped in our previous report [15], seven markers (beginning with letters “CB” or “CF”) derived from first genomic SSR libraries [4], two markers (DCF115, DCB140) developed by Smulders et al. [17], and DCEST07 derived from publicly available ESTs. Of these, 130 were genomic SSR markers and the other 282 were EST-SSR markers.

### Refined map construction for 85P population

**Figure 1:**
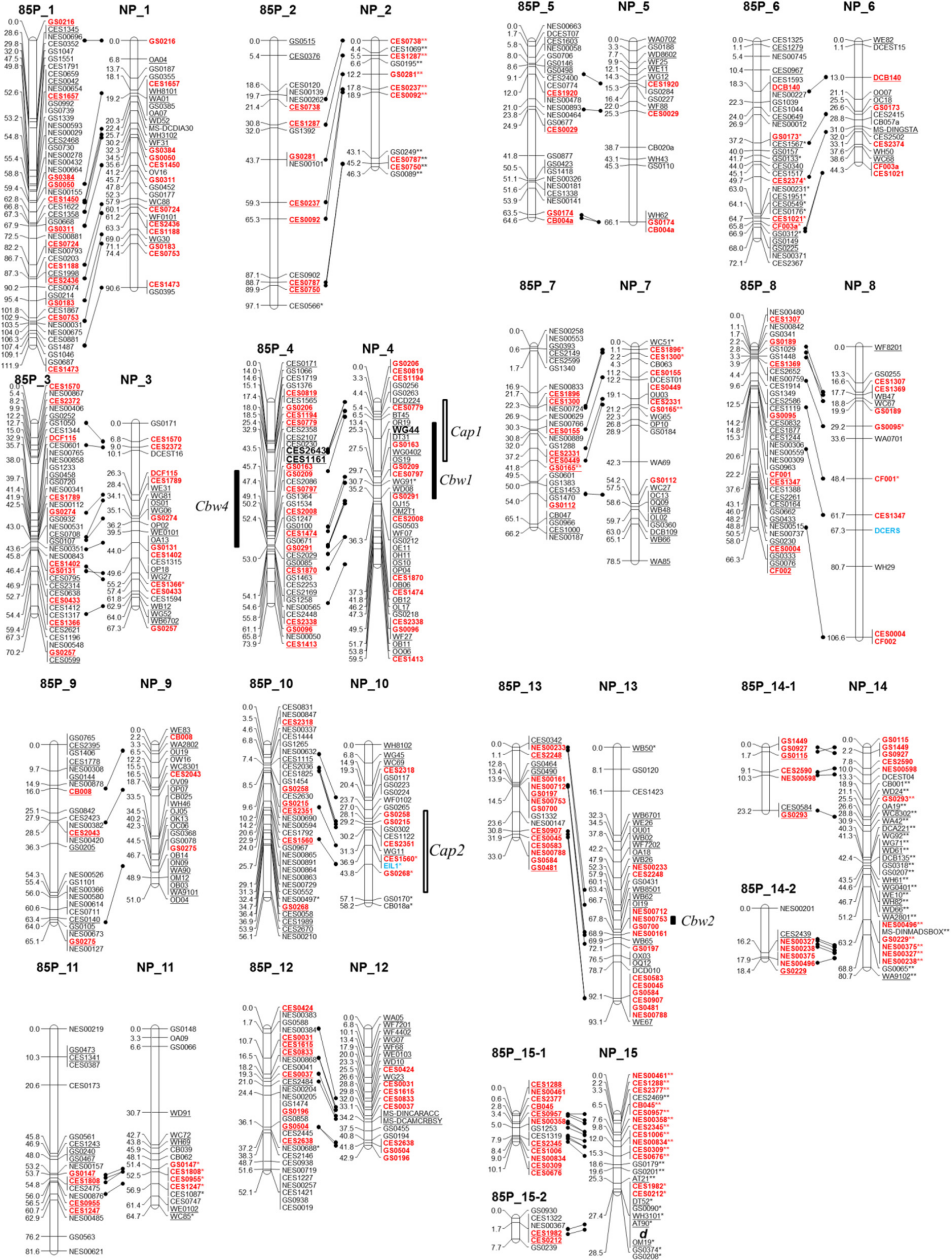
**Construction of genetic linkage maps and QTL analysis for bacterial wilt (CBW) resistance and anthocyanin pigmentation contents in petals (CAP) in carnation.** Two populations were used for the 85P population, which was derived from a cross between line 85–11 and ‘Pretty Favvare’, and the NP population, which was derived from a cross between ‘Carnation Nou No.1’ and ‘Pretty Favvare’. Linkage groups (LGs) were designated as 85P_1 to 85P_15-2 for the 85P map, and as NP_1 to NP_15 for the NP map. Genetic distances (cM) and SSR loci are listed on the left and right sides of each LG, respectively. Distorted segregation is indicated by a significant *P* value in the chi-square test: ^*^, *P* < 0.05; ^**^, *P* < 0.01. Published loci previously reported by Yagi et al. [14,15] are underlined. SSR loci common to both maps are indicated in red and connected with lines. STS loci designed by De Benedetti et al. [20] are shown in blue. Boxes indicate QTL regions with LOD values greater than thresholds (in Table 3). Tightly linked markers identified in previous reports [14,15] are indicated in bold. Single flower locus in Onozaki et al. [18] is shown as “d” on LG NP_15.

Linkage analysis using a minimum LOD score of 7.0 revealed 17 LGs (Figure 1, Table 1). This result indicated the existence of several minor LGs consisting of a small number of loci within less than 50 cM. To clarify the relationships among the minor LGs, we transferred all SSR loci in the five minor LGs (eventual LGs: 85P_13, 14–1, 14–2, 15–1, and 15–2) to the “NP” map updated from the “A” map described in Yagi et al. [14] by adding RAPD and SSR loci (Figure 1, Table 1). Thirteen SSR loci out of 18 loci on LG 85P_13 (corresponding to B13 in Yagi et al. [15]) were mapped to lower regions of LG NP_13 (corresponding to A2 in Yagi et al. [14]). Six SSR loci out of seven loci on LG 85P_14-1 (corresponding to B14 in Yagi et al. [15]) and five out of seven loci on LG 85P_14-2 (corresponding to B16 in Yagi et al. [15]) were mapped to LG NP_14 (corresponding to A3 in Yagi et al. [14]). Eleven out of 13 loci on LG 85P_15-1 (corresponding to B15 in Yagi et al. [15]) and two out of six loci on LG 85P_15-2 (no linkage group in Yagi et al. [15]) mapped to LG NP_15 (corresponding to A16 in Yagi et al. [14] and Onozaki et al. [18]). The positions of all SSR loci mapped to both LGs were conserved between LGs. These results indicated that four of the five minor LGs could be integrated into two LGs. Consequently, the number of LGs corresponded to the basic chromosomal number in carnation (x = 15).

The LG names and their order on the 85P map follow those used in a previous report [15] (Table 1). The total length of the 85P map was 969.6 cM, with an average distance of 2.4 cM between loci. The LGs ranged in size from 7.7 cM (LG 85P_15-2) to 111.9 cM (LG 85P_1), with an average length of 57.0 cM. The number of loci mapped per LG ranged from 6 (LG 85P_15-2) to 48 (LG 85P_1), with an average of 24.2. The marker density (loci/cM) ranged from 0.16 (85P_2) to 1.29 (85P_15-1), with an average of 0.49. The entire marker density was 0.42. Two published but previously unmapped loci, CES0212 and CES1982, were mapped onto LG 85P_15-2 in this study (Figure 1). Of the 412 SSR loci, 397 showed segregation patterns that fitted the expected segregation ratio of 1:2:1, while the segregations of 14 loci (*P* < 0.05) and 1 locus (*P* < 0.01) differed from the expected ratio. Most of the loci showing different segregation ratios were located on LG 85P_ 6 (*n* = 11); the others were on LGs 85P_ 2, 7, 10, and 12.

### Comparison of LGs between two maps using SSR markers

To reveal the relationships for other LGs between the 85P and NP maps, more than five SSR loci in each LG of the 85P map were tested in the refined NP map. This analysis revealed that at least three loci were connected to the homologous LG (Figure 1, Table 1). The 85P and NP maps had 125 loci in common. The map position of each SSR locus showed good collinearity between homologous LGs. We identified 635 loci in carnation using the two linkage maps.

### Refined map construction for NP population

The NP map was significantly improved from our previous report [14]. We constructed the NP map using 94 lines derived from a cross between ‘Carnation Nou No. 1’ and ‘Pretty Favvare’. Linkage analysis using a minimum LOD score of 7.0 revealed 15 LGs consisting of 348 loci covering 978.3 cM (Figure 1, Table 2). The LG names on the NP map were rearranged to be consistent with homologous LGs on the 85P map. We mapped 192 new SSR markers that showed single-locus polymorphisms onto the NP map. Of those, 95 SSR loci were derived from the genomic sequence; 80 were “GS” SSR loci derived from the SSR-enriched genomic library developed by Yagi et al. [15], 14 were “CB” or “CF” loci derived from the SSR-enriched genomic library developed by Kimura et al. [4], and DCD010 on LG NP_13 was developed by Smulders et al. [17]. The other 97 SSR loci were derived from EST information; MS-DCDIA30 on LG NP_1 and MS-DINMADSBOX on LG NP_14 were developed by Smulders et al. [19], 78 were “CES” SSR loci derived from Sanger-based EST analysis [15], four were “DCEST” SSR loci derived from public EST information, and 13 were “NES” SSR loci derived from RNA-seq analysis using NGS technology. These newly mapped SSR loci for the NP map are shown in Additional file 2.

**Table 2:** Number and type of loci, genetic distance and marker density for the refined NP map

**New LG**	**Previous LG (Yagi et al. **[14]**)**	**Total loci**	**Published RAPD loci (in Yagi et al. **[14]**)**	**New RAPD and STS loci**	**Published SSR loci (in Yagi et al. **[14]**)**	**New SSR loci**							**Length of LGs (cM)**	**Marker density (loci/cM)**
						**SSR locus names**						**Subtotal**		
						**GS-**	**MS-, DCD-**	**CB-, CF-**	**DCEST-**	**CES-**	**NES-**			
					**SSR resource**									
					**Genomic, EST**	**Genomic**	**Genomic, EST**	**Genomic**	**EST**	**EST**	**EST**			
NP_1	A7, A10	30	10	1	0	11	1	0	0	7	0	19	90.6	0.33
NP_2		11	0	0	0	4	0	0	0	7	0	11	46.3	0.24
NP_3	Lower region of A1	26	12	0	1	4	0	0	1	8	0	13	67.3	0.39
NP_4	A6	41	21	0	1	10	0	0	0	9	0	19	59.5	0.69
NP_5	A4	17	8	0	0	5	0	2	0	2	0	9	66.1	0.26
NP_6	Upper region of A1	15	5	0	2	1	0	2	1	4	0	8	44.3	0.34
NP_7	A5	24	11	1	1	4	0	1	1	5	0	11	78.5	0.31
NP_8	A12	15	3	3	0	3	0	2	0	4	0	9	106.6	0.14
NP_9	A8	24	17	1	0	3	0	2	0	1	0	6	51.0	0.47
NP_10	A9	20	4	2	0	9	0	1	0	4	0	14	58.2	0.34
NP_11	A13, A15	16	5	1	0	3	0	2	0	5	0	10	64.7	0.25
NP_12	A11, A14	20	8	0	2	4	0	0	0	6	0	10	42.9	0.47
NP_13	A2	32	15	0	0	6	1	0	0	5	5	17	93.1	0.34
NP_14	A3	33	13	1	2	8	1	1	1	1	5	17	80.7	0.41
NP_15	A16	24	5	0	0	5	0	1	0	10	3	19	28.5	0.84
Total		348	137	10	9	80	3	14	4	78	13	192	978.3	0.36

Eight new RAPD markers (beginning with the letters “O” or “W”) were mapped onto LGs NP_1, 7, 8, 9, 10, 11, 14 (Figure 1, Table 2). We tested 22 ethylene biosynthesis and response pathway gene-specific sequence-tagged site (STS) primers designed by De Benedetti et al. [20]. Among these 22 STS markers, one derived from *DCERS* was mapped onto LG NP_8 and one derived from *EIL1* (ethylene insentive3-like protein1) was mapped onto LG NP_10. Both of these genes encode putative ethylene receptors.

In total, 201 SSR, 145 RAPD, and two STS loci were mapped onto the NP map. The total length of the map was 978.3 cM, with an average distance of 2.8 cM between loci (Table 2). Linkage groups ranged in size from 28.5 cM (LG NP_15) to 90.6 cM (LG NP_1), with an average length of 65.2 cM. The number of loci mapped per LG ranged from 11 (LG NP_2) to 41 (LG NP_4), with an average of 23.2. The marker density (loci/cM) ranged from 0.14 (85P_8) to 0.84 (NP_15), with an average of 0.39. The entire marker density was 0.36. Of the 348 loci, the segregation of 266 loci fitted the expected 1:1 ratio, while the segregations of 28 (*P* < 0.05) and 54 (*P* < 0.01) loci deviated from the expected ratio (Figure 1). Most of the deviated loci were located on LG NP_ 2, 14, and 15.

### Mapping of QTLs and a flower-type locus

We mapped QTLs onto the two maps by analyzing data reported previously [14,15,21]. The mapped QTLs are shown in Figure 1 and listed in Table 3. A major QTL for CBW resistance derived from line 85–11 (*Cbw4*, LOD = 24.3) was mapped onto LG 85P_4. There were no other QTLs with an LOD score above 3.7, which was the minimum LOD score after 1000 permutation tests [22,23]. A new marker linked to *Cbw4*, CES0230, was identified in this analysis, in addition to the *Cbw4*-linked markers CES1161 and CES2643 identified previously [15]. For the NP map, QTLs for CBW resistance derived from *D. capitatus ssp. andrezejowskianus* were mapped in our previous study [14]. *Cbw1* and *Cbw2* were mapped onto LG NP_4 (LOD = 22.1) and LG NP_13 (LOD = 2.8), respectively. The nearest marker to *Cbw1* was WG44, the same result obtained previously [14,24]. There were no other significant QTLs with an LOD score above 2.7, which was the minimum LOD score after 1000 permutation tests, hence, *Cbw3*, which had a low LOD score of 2.3 in our previous study [14], was not detected in this study. Flower color is an important trait in ornamentals. Therefore, we identified QTLs related to the anthocyanin content in petals (carnation anthocyanin pigmentation loci [*Cap*]) in our previous study [15]. *Cap1* (LOD = 6.1) and *Cap2* (LOD =5.3) were mapped onto LG NP_4 and LG NP_10, respectively. We also mapped a phenotypic locus for flower-type. We identified a single flower-type locus in a previous study [18]. The single flower-type locus derived from *D. capitatus ssp. andrezejowskianus* was mapped onto LG NP_15. In addition to the four co-segregating RAPD markers identified previously, four SSR markers (CE1982, CES0212, GS0090, and GS0374) were identified in this study.

**Table 3:** QTL analysis for bacterial wilt resistance (CBW) and flower anthocyanin pigmentation contents (CAP) in carnation

**QTL**	**LG**	**Peak position (cM)**	**Adjacent marker**		**LOD score**	**Phenotypic variance (%)**	Threshold value*
			**Left**	**Right**			
*Cbw4*	85P_4	44.0	CES1161	GS0163	24.3	63.5	3.7
*Cbw1*	NP_4	21.9	OR19	WG44	22.1	59.0	2.7
*Cbw2*	NP_13	63.4	WB8501	WB62	2.8	4.8	2.7
*Cap1*	NP_4	6.5	BT45	OR19	6.1	19.7	2.7
*Cap2*	NP_10	24.2	WF0102	GS0265	5.3	17.2	2.7

*Thresholds were determined by 1,000 permutation tests using WinQTL cartographer software.

## Discussion

High-density linkage maps are useful for locating genes of interest for marker-assisted selection and to identify QTLs. In this study, we revised previously constructed linkage maps to add new markers and increase their resolution. In total, 412 single-locus SSR markers are mapped, 234 of which are new markers. We have mapped a large number of SSR markers in the linkage map of carnation, which is not a model crop and had few available SSRs. Recently, NGS has been used to develop several types of genetic markers such as restriction site associated DNA (RAD) and single nucleotide polymorphism (SNP) markers, including SSR markers [25-29]. One full NGS shotgun run can yield thousands of genome-wide SSR markers. We obtained 1,162,126 high-quality cDNA reads by 454 pyrosequencing technology and finally designed 4,177 SSR primer pairs [16]. We mapped 107 single-locus SSR markers (11.9%) of 897 tested SSR primer sets obtained from NGS analysis. The success ratio was similar to that obtained using “CES” markers derived from Sanger-sequencing EST analysis in this mapping population (11.3%; 174/1530 markers). NGS technologies allow the efficient identification of large numbers of SSR loci at a fraction of the cost and effort of traditional SSR isolation approaches [28]. Therefore, these technologies will increase the amount of genome information and improve the quality of genomic resources for many plant species, especially ornamental species that tend to have complex genome structures (e.g. polyploidy) [29].

The ratio of distorted loci was larger in the NP map (23.6%; 82/348 loci) than in the 85P map (3.6%; 15/412 loci). Most of the distorted loci were located on LG 85P_ 6 (*n* = 11) on the 85P map, but the corresponding LG on the NP map, NP_6, had no distorted loci (Figure 1). For the NP map, most of the loci located on NP_2, 14, 15 were distorted. Thus, the regions of distortion differed between the two linkage maps. Distorted segregations in linkage maps have been reported for other ornamentals, including *Rhododendron*[30], diploid rose [31-34], Asiatic hybrid lily [35], and Japanese gentian [27]. The causes of segregation distortion have been suggested to include aneuploidy, chromosomal translocation, competition among gametes, and the inheritance of alleles affecting the viability of the zygote, embryo, or seedling [1,36,37]. ‘Pretty Favvare’ is the common mapping parent for both maps, therefore, discordance between normal carnation and ‘Carnation Nou. No.1’, which is derived from an interspecific cross, may account for the higher ratio of distorted loci on the NP map.

Linkage analysis for the 85P map revealed 17 LGs. Comparative analysis between LGs using SSR loci in the updated NP map revealed that four minor LGs could be integrated into two LGs. For the NP map, the addition of a large number of SSR markers converged the map to 15 LGs. Homologous LGs from different populations were identified using the same anchored SSR loci. These results showed that the present LGs would correspond to the basic chromosome number in carnation (x = 15, [7]). Even though 234 SSR loci were added to the 85P map, the total map length was only 126.0 cM longer than that of the previously constructed map (178 SSR loci, 843.6 cM, [15]) and many markers were mapped to the same locus (Figure 1). This might be because of low recombination frequencies in this mapping population, or homozygous chromosomal regions between parental lines. In a linkage map for pear, the short coverage of LGs revealed that large regions of LGs were homozygous [38]. Thus, these findings might suggest a limitation for constructing a saturated linkage map using this population.

We analyzed previously reported genotype and phenotype data to map QTLs for CBW and flower color pigmentation, and the flower-type locus, onto the refined maps. This allowed us to identify some new markers linked to those traits. As well as RAPD markers, co-dominant SSR markers linked to the flower-type locus would be useful, although further analyses are needed to assess their effectiveness as selection markers. *Cbw3* (with a small LOD score of 2.3 in our previous study [14]), was not detected in this study. This was probably because of the change in analytical method, i.e., from interval mapping using MAPMAKER software [39] to composite interval mapping (CIM, [40,41]) using WinQTL Cartographer [42]. Moreover, the expansion and rearrangement of the map would also affect the ability to detect this locus. Our revised maps described in this paper will allow more precise QTL analysis, including identification of loci associated with various attributes such as flower vase life, an important trait for ornamental plants that has not yet been mapped in the mapping population.

We identified a total of 635 loci including 488 SSR in carnation using the two linkage maps. We believe that these linkage maps will serve as reference genetic linkage maps for carnation and that many of the mapped SSR markers will be useful to compare these maps with others constructed using data from different mapping populations. Currently, we are producing another mapping population and will construct a new linkage map. Moreover, whole genome sequencing by the NGS system is underway.

## Conclusions

We improved two carnation linkage maps and mapped a large number of SSR loci. The genetic linkage map derived from a cross between the CBW-resistant line 85–11 and susceptible ‘Pretty Favvare’ comprising 412 SSR loci covering 969.6 cM, with an average distance of 2.4 cM between loci. The genetic linkage map derived from a cross between CBW-resistant ‘Carnation Nou No.1’ and susceptible ‘Pretty Favvare’ comprised 201 SSR, 145 RAPD, and two STS loci covering 978.3 cM, with an average 2.8 cM distance between loci. These two refined maps were anchored with 125 SSRs loci. We identified 635 loci in carnation using the two linkage maps. The number of LGs was coincident with the haploid chromosome number of carnation (x = 15). Our genetic linkage maps, combined with the SSR markers developed in this study, represent reference genetic linkage maps for members of the genus *Dianthus*, including carnation, which will be useful for mapping QTLs associated with various traits, and for improving elite carnation cultivars in breeding programs.

## Methods

### Plant materials

We used 91 F_2_ progenies of the line 85–11 and ‘Pretty Favvare’ for SSR-based map construction. To develop useful SSR markers for carnation cultivars derived from closely related crosses, we improved this mapping population to construct a higher density linkage map. The standard-type carnation line 85–11 is highly resistant to CBW and the spray-type carnation cultivar ‘Pretty Favvare’ is susceptible to CBW. Line 85–11 was selected from the third-generation lines in our breeding program for improving flower vase life, and showed low ethylene production in whole flowers during senescence [43]. Of the 90 F_2_ progenies used in our previous study [15], one progeny was excluded and two new progenies were added in this study. This population was renamed as the 85P population.

To compare the LGs with those in our RAPD-based map [14], 94 lines from 134 progenies from previous mapping populations were used for map construction. This population was renamed as the NP population. The NP population was derived from a cross between the CBW-resistant cultivar, ‘Carnation Nou No.1’, and the susceptible carnation cultivar, ‘Pretty Favvare’. ‘Carnation Nou No.1’ is an interspecific hybrid between a susceptible *D. caryophyllus* ‘Super Gold’ and the highly resistant wild species, *D. capitatus* ssp. *andrzejowskianus*[44]. The segregating population was obtained by crossing ‘Pretty Favvare’, a susceptible *D. caryophyllus*, with ‘Carnation Nou No.1’. Therefore, the segregating progenies represent a backcross (BC_1_) population. The marker loci derived from *D. capitatus* ssp. *andrzejowskianus* were genotyped for map construction in this study.

### SSR marker analysis

To improve the 85–11 map, we screened for polymorphisms between parents using 1,013 “GS” and 897 “NES” primer pairs. We also rescreened for polymorphisms using 1,530 “CES” primer pairs using fluorescent fragment analysis instead of electrophoresis on polyacrylamide gels. Polymorphic markers between the parents were used for mapping in the progeny. To improve the NP map, we screened for polymorphisms between parents using the SSR primer pairs used in Yagi et al. [15], including 539 “GS”, 50 “CB”, 4 “CF”, 5 “MS”, 11 “DC” and 31 “DCEST” SSR primer pairs.

The PCR conditions and the subsequent analyses were as described by Yagi et al. [15]. The reactions were run on an ABI PRISM 3730 automated DNA sequencer (Life Technologies Co., Carlsbad, CA, USA). The sizes of the amplified bands were determined from a DNA internal standard 400HD-ROX (Life Technologies Co.) using Gene Mapper software (Life Technologies Co.).

### RAPD and STS analysis for NP map

RAPD analysis was performed according to the methods of Yagi et al. [14]. We tested 22 ethylene biosynthesis and response pathway gene-specific STS primers designed by De Benedetti et al. [20]. PCRs were carried out in a reaction volume 10 μL, containing 1 × Ex Taq buffer, 10 ng of genomic DNA, 0.2 mM dNTPs, 1 pmol forward and reverse primers and 0.25 units Ex Taq DNA polymerase (Takara Bio Inc., Otsu, Japan). Amplifications were carried out in a Takara PCR Thermal Cycler Dice (Takara Bio Inc.). The temperature conditions for PCR were as follows: 94°C for 1 min; 30 cycles at 94°C for 30 s, 55°C for 30 s, 72°C for 1 min; and 7°C for 5 min; followed by cooling at 4°C to stop the reaction. The amplified products were separated by electrophoresis at 100 V for 40 min on a 1.5% agarose gel.

### Construction of a genetic linkage map

We constructed the genetic linkage map using JoinMap ver. 4.1 (Kyazma B. V., Wageningen, Netherlands). Marker data were assigned to the LGs using a minimum LOD score of 7.0. The linkage groups were displayed using version 2.2 of MapChart [45]. We used the Kosambi mapping function [46] to calculate the genetic distance between markers. Segregation distortion was determined by calculating chi-square values for all mapped markers using JoinMap ver. 4.1 (Kyazma). Significance thresholds of *P* < 0.05 and *P* < 0.01 were plotted as asterisks on the graphs. Construction of the NP map revealed that LG A1 in Yagi et al. [14] was divided into two groups (LG NP_3 and NP_6) and the previously identified LGs (A7 and A10, A13 and A15, A11 and A14) were integrated into new LGs; NP_1, NP_11 and NP_12, respectively (Table 2).

### Comparing LGs using SSR markers

All SSR loci located on LGs with a small number of loci on the 85P map (85P_13, 14–1, 14–2, 15–1 and 15–2) were transferred onto the NP map. More than five SSR loci in each LG on the 85P map were tested on the NP map.

### QTL analysis

Genotype data and phenotype data from previous studies [14,15,21] were analyzed to map QTLs using the CIM method [40,41], as implemented in version 2.5 of WinQTL Cartographer [42]. The CIM analysis was performed using Model 6 software, scanning the genetic map, and estimating the likelihood of a QTL and its corresponding effects at intervals of 0.5 cM. Significant marker cofactors were used to adjust the phenotypic effects associated with other positions on the genetic map. The number of marker cofactors for the background control was set by forward–backward stepwise regression. Thresholds were determined by means of permutation tests [22,23], using 1000 permutations and a significance level of 0.05.

## Competing interests

The authors declare that they have no competing interests.

## Authors’ contributions

MY participated in the design of the experiments, developed and genotyped the SSR, RAPD, and STS markers, constructed the maps, interpreted the data, and wrote the paper. TY participated designing the experiments and revised the manuscript. SI, ST, and HH developed and screened SSR markers from Sanger-sequenced ESTs and developed SSR markers from RNA-seq. KT collected RNA-seq data and grew the plants with MY and TO. TO participated in designing the experiments, grew the plants, and revised the manuscript. HY grew the plants and participated in the discussion of the results and helped to revise the manuscript. All the authors read and approved the manuscript.

## Supplementary Material

Additional file 1 List of new SSR markers mapped onto 85P map. Click here for file

Additional file 2 List of new SSR markers only mapped onto NP map. Click here for file
